# A Novel Multiparametric Approach to 3D Quantitative MRI of the Brain

**DOI:** 10.1371/journal.pone.0134963

**Published:** 2015-08-18

**Authors:** Giuseppe Palma, Enrico Tedeschi, Pasquale Borrelli, Sirio Cocozza, Carmela Russo, Saifeng Liu, Yongquan Ye, Marco Comerci, Bruno Alfano, Marco Salvatore, E. Mark Haacke, Marcello Mancini

**Affiliations:** 1 Institute of Biostructure and Bioimaging, National Research Council, Naples, Italy; 2 Department of Advanced Biomedical Sciences, University of Naples Federico II, Naples, Italy; 3 IRCCS SDN, Naples, Italy; 4 The MRI Institute for Biomedical Research, Waterloo, ON, Canada; 5 Department of Radiology, Wayne State University, Detroit, MI, United States of America; 6 The MRI Institute for Biomedical Research, Detroit, MI, United States of America; University of Ulm, GERMANY

## Abstract

Magnetic Resonance properties of tissues can be quantified in several respects: relaxation processes, density of imaged nuclei, magnetism of environmental molecules, etc. In this paper, we propose a new comprehensive approach to obtain 3D high resolution quantitative maps of arbitrary body districts, mainly focusing on the brain. The theory presented makes it possible to map longitudinal (*R*_1_), pure transverse (*R*_2_) and free induction decay ($R_{2}^{*}$) rates, along with proton density (PD) and magnetic susceptibility (*χ*), from a set of fast acquisition sequences in steady-state that are highly insensitive to flow phenomena. A novel denoising scheme is described and applied to the acquired datasets to enhance the signal to noise ratio of the derived maps and an information theory approach compensates for biases from radio frequency (RF) inhomogeneities, if no direct measure of the RF field is available. Finally, the results obtained on sample brain scans of healthy controls and multiple sclerosis patients are presented and discussed.

## 1 Introduction

Multi-parametric quantitative Magnetic Resonance Imaging (qMRI) based on the relaxometry properties of intracranial tissues has long been and still is an active field of research in medicine and physics [1]. Several approaches have been used, aiming at obtaining quantitative estimates of the longitudinal relaxation rate (*R*_1_), transverse relaxation rate (*R*_2_) and proton density (PD) of brain tissues [2–7]. Other relaxation parameters whose MR signals can be usefully exploited to discriminate the microstructure of brain tissues are the free induction decay (FID) rate ($R_{2}^{*}$) [4] and the magnetic susceptibility (*χ*) [8]. In this paper, we present a new acquisition and processing procedure that, starting from a set of conventional high resolution 3D Steady State sequences, makes it possible to achieve full brain coverage for absolute measurements of intracranial compartments. For the first set of tissue properties, Radio Frequency (RF) *B*_1_ inhomogeneities can create problems. As pointed out in [9], a 3D acquisition protocol is preferred to remove intra-voxel biases, known to arise from imperfect 2D RF-pulse profiles that cause different isochromat evolutions in response to different effective flip angles.

Here, we prove that a set of steady-state sequences, acquired with variable flip angles and different phase coherence, makes it possible to derive quantitative volumetric *R*_1_, *R*_2_, PD, $R_{2}^{*}$ and *χ* maps of the brain tissues. We also show that high Signal-to-Noise Ratio (SNR) full brain coverage with a sub-millimeter resolution may be obtained in a total acquisition time of 14 minutes. The proposed solution for the relaxation rates is fully analytic and allows for the inversion of the signal equations for *R*_1_, *R*_2_, PD and $R_{2}^{*}$ maps in a typical 3D dataset within a few seconds (5 s for a 320 × 270 × 128 voxel array on a 2.7 GHz Intel Core i5 processor).

The plan of the paper is as follows. In §2, the Bloch equations of the sequences are briefly reviewed; then, the proposed mathematical scheme to derive *R*_1_, *R*_2_, PD, $R_{2}^{*}$ and *χ* maps is presented in full detail. In §3, we provide a description of a sample experimental setup performed to prove the feasibility of the general theoretical scheme of §2. Finally, results are presented in §4 and discussed in §5.

## 2 Theory

### 2.1 Sequence Bloch equations

Within the steady-state sequence family, we selected the spoiled Gradient Echo (GRE) and the balanced Steady-State Free-Precession (bSSFP) sequences because of their low sensitivity to flow artifacts in both CSF in ventricles and blood within vessels.

The complex signal of a spoiled GRE sequence is

$$\begin{array}{c} S_{FL}=K\cdot M_{0}\cdot \sin\theta \cdot \frac{1-E_{1}}{1-E_{1}\cos\theta }\cdot e^{-T_{E}\cdot \left(R_{2}^{*}+i\gamma_{n}\Delta B\right)+i\phi_{0}}\;, \end{array}$$ (1)

where *K* is the complex coil sensitivity, *M*_0_ is the equilibrium magnetization, *θ* is the flip angle, *E*_1_ ≡ exp(−*T*_*R*_ ⋅ *R*_1_) (*T*_*R*_ being the repetition time), *T*_*E*_ is the echo time, *γ*_n_ is the gyromagnetic ratio of the imaged nucleus, Δ*B* is the local magnetic field variation and *ϕ*_0_ is the phase-shift induced by the RF-pulse.

The complex signal of a bSSFP sequence is

$$\begin{array}{c} S_{TF}=K\cdot M\cdot \frac{1-E_{2}\cdot e^{-i\varphi }}{1-a\cos\varphi }e^{-T_{E}\cdot \left(R_{2}+i\tilde{\varphi }/T_{R}\right)}\;, \end{array}$$ (2)

where

$$\begin{array}{c} M\equiv iM_{0}\cdot \frac{(1-E_{1})\sin\theta }{b}\;; \end{array}$$ (3)

$$\begin{array}{c} a\equiv E_{2}\cdot \frac{\left(1-E_{1}\right)\left(1+\cos\theta \right)}{b}\;; \end{array}$$ (4)

$$\begin{array}{c} b\equiv 1-E_{1}\cos\theta -\left(E_{1}-\cos\theta \right)E_{2}^{2}\;; \end{array}$$ (5)

*E*_2_ ≡ exp(−*T*_*R*_ ⋅ *R*_2_), *φ* = *γ*_n_Δ*BT*_*R*_ + Δ*φ* is the resonance offset angle and $\tilde{\varphi }$ depends on how the phase-cycling Δ*φ* is achieved: if the frequency of the Larmor reference frame is formally shifted, then $\tilde{\varphi }=\varphi$; otherwise, if a constant phase term is added to the previous RF phase each time a new RF pulse is applied, then $\tilde{\varphi }=\gamma_{\text{n}}\Delta BT_{R}$.

### 2.2 Band-free bSSFP reconstruction

Due to the dependence of (Eq 2) on *φ*, bSSFP images are well known to be affected by severe banding artifacts, particularly in regions with rapidly varying susceptibility or main magnetic field [9]. To avoid a biased *R*_2_-map, in the following a new dataset *S*_0_ (independent of *φ*) is derived from a set of *n* bSSFPs (*n* ≥ 3) acquired with increasing resonance offset angles (phase cycling) as described in [10, 11].

The *φ*-independent contrast *S*_0_ we aim to derive corresponds to

$$\begin{array}{c} S_{0}\equiv K\cdot M\cdot e^{-T_{E}\cdot R_{2}}\;. \end{array}$$ (6)

For each phase-cycling *j* ∈ {1, …, *n*}, we can formally introduce

$$\begin{array}{ccc} {\tilde{S}}_{j} & \equiv & S_{TF,j}e^{-i\left({\tilde{\varphi }}_{j}/T_{R}-\gamma_{n}\Delta B\right)T_{E}}\;, \end{array}$$ (7)

$$\begin{array}{ccc} \alpha & \equiv & S_{0}e^{i\varphi T_{E}/T_{R}}\;, \end{array}$$ (8)

$$\begin{array}{ccc} \beta & \equiv & S_{0}E_{2}e^{i\varphi (T_{E}/T_{R}-1)}\;, \end{array}$$ (9)

$$\begin{array}{ccc} \gamma & \equiv & ae^{i\varphi }\;. \end{array}$$ (10)

Defining

$$A\equiv {\left[\begin{array}{ccccc} 1 & 0 & \cdots & 1 & 0\\ 0 & 1 & \cdots & 0 & 1\\ -\cos(\Delta \varphi_{1}) & \sin(\Delta \varphi_{1}) & \cdots & -\cos(\Delta \varphi_{n}) & \sin(\Delta \varphi_{n})\\ -\sin(\Delta \varphi_{1}) & -\cos(\Delta \varphi_{1}) & \cdots & -\sin(\Delta \varphi_{n}) & -\cos(\Delta \varphi_{n})\\ ℜ({\tilde{S}}_{1})\cos(\Delta \varphi_{1}) & ℑ({\tilde{S}}_{1})\cos(\Delta \varphi_{1}) & \cdots & ℜ({\tilde{S}}_{n})\cos(\Delta \varphi_{n}) & ℑ({\tilde{S}}_{n})\cos(\Delta \varphi_{n})\\ -ℜ({\tilde{S}}_{1})\sin(\Delta \varphi_{1}) & -ℑ({\tilde{S}}_{1})\sin(\Delta \varphi_{1}) & \cdots & -ℜ({\tilde{S}}_{n})\sin(\Delta \varphi_{n}) & -ℑ({\tilde{S}}_{n})\sin(\Delta \varphi_{n}) \end{array}\right]}^{T}\;,$$ (11)

$$\begin{array}{c} y\equiv [\begin{array}{c} ℜ({\tilde{S}}_{1})\\ ℑ({\tilde{S}}_{1})\\ \vdots \\ ℜ({\tilde{S}}_{n})\\ ℑ({\tilde{S}}_{n}) \end{array}]\;\text{and}\;x\equiv [\begin{array}{c} ℜ(\alpha )\\ ℑ(\alpha )\\ ℜ(\beta )\\ ℑ(\beta )\\ ℜ(\gamma )\\ ℑ(\gamma ) \end{array}] \end{array}$$ (12)

from Eqs (2)–(10) it can be shown that

$$\begin{array}{c} y=A\cdot x\;. \end{array}$$ (13)

If *n* ≥ 3, the Moore-Penrose pseudoinverse of **A** provides the least-squares estimate of the unknown variables *α*, *β* and *γ* (cast into **x** form) as a function of the bSSFP datasets (cast into **y** form—each ${\tilde{S}}_{j}$ is known and corresponds to the acquired scan multiplied by a function of the acquisition parameters):

$$\begin{array}{c} x={\left(A^{T}A\right)}^{-1}A^{T}\cdot y\;. \end{array}$$ (14)

From Eqs (8)–(9), it follows that

$$\begin{array}{c} \varphi =∠\frac{\alpha }{\beta }\;, \end{array}$$ (15)

whence, from (Eq 8), we obtain

$$\begin{array}{c} S_{0}=\alpha e^{-i\varphi T_{E}/T_{R}}\;. \end{array}$$ (16)

### 2.3 *R*_1_-map

The acquisition of two or more spoiled GRE scans performed at different flip angles and fixed *T*_*R*_ makes it possible to estimate *R*_1_ [9]. The magnitude of (Eq 1) satisfies

$$\begin{array}{c} \frac{\left|S_{FL}\right|}{\sin\theta }=E_{1}\frac{\left|S_{FL}\right|}{\tan\theta }+|K|M_{0}\left(1-E_{1}\right)e^{-T_{E}\cdot R_{2}^{*}}\;. \end{array}$$ (17)

(Eq 17) represents the equation of a straight line with slope *E*_1_ in a plane whose coordinate pairs are (*S*_*FL*_/tan *θ*, *S*_*FL*_/sin *θ*). Therefore, if we write the signal intensity of the *j*-th echo (*j* ∈ {1, …, *m*}, *m* ≥ 2) of the *i*-th spoiled GRE acquisition (*i* ∈ {1, …, *l*}, *l* ≥ 2) as

$$\begin{array}{c} S_{i,j}=M_{i}\cdot \exp\left(-T_{E,j}\cdot R_{2}^{*}\right)\;, \end{array}$$ (18)

where

$$\begin{array}{c} M_{i}=|K|M_{0}\cdot \sin\theta_{i}\cdot \frac{1-E_{1}}{1-E_{1}\cos\theta_{i}}\;, \end{array}$$ (19)

*R*_1_ can be estimated via simple linear regression of (Eq 17) as

$$\begin{array}{c} R_{1}=\frac{1}{T_{R}}\cdot \log\frac{\text{Var}[\{S_{i}/\tan\theta_{i}\}]}{\text{Cov}[\{S_{i}/\tan\theta_{i}\},\{S_{i}/\sin\theta_{i}\}]}\;, \end{array}$$ (20)

with

$$\begin{array}{c} S_{i}=\sqrt{\sum_{j=1}^{m}S_{i,j}^{2}}\;. \end{array}$$ (21)

### 2.4 $R_{2}^{*}$-map

If we define

$$\begin{array}{c} s_{j}\equiv \sqrt{\sum_{i=1}^{l}S_{i,j}^{2}}\;, \end{array}$$ (22)

from (Eq 18) it follows that

$$\begin{array}{c} s_{j}=\sqrt{\sum_{i=1}^{l}M_{i}^{2}}\cdot \exp\left(-T_{E,j}\cdot R_{2}^{*}\right)\;, \end{array}$$ (23)

whence $R_{2}^{*}$ turns out to be the linear coefficient between $\text{log}s_{j}^{-1}$ and *T*_*E*,*j*_. A simple linear regression yields therefore to

$$\begin{array}{c} R_{2}^{*}=-\frac{\text{Cov}[\{T_{E,j}\},\{\log s_{j}\}]}{\text{Var}[\{T_{E,j}\}]}\;. \end{array}$$ (24)

### 2.5 PD-map

Once both *R*_1_ and $R_{2}^{*}$ maps are known, it is also possible to provide an estimate of proton density (in arbitrary units) by inverting (Eq 18) for each *i*, *j* with respect to |*K*|*M*_0_ from (Eq 19), and averaging the results over the acquired echoes:

$$\begin{array}{c} |K|M_{0}=\frac{\sum_{i,j}[S_{i,j}^{2}\cdot \frac{1-E_{1}\cos\theta_{i}}{\left(1-E_{1}\right)\sin\theta_{i}}\cdot e^{T_{E,j}\cdot R_{2}^{*}}]}{\sum_{i,j}S_{i,j}}\;. \end{array}$$ (25)

### 2.6 *χ*-map

An extensive description of the susceptibility quantitation techniques is reported in [8]. Here we briefly summarize the actually adopted strategy.

The net magnetic field **B**(**r**) resulting from the magnetization **M** induced within a matter distribution by an external magnetic field **B**_0_ is given by

$$\begin{array}{c} B\left(r\right)=B_{0}+\frac{\mu_{0}}{4\pi }\int_{V}d^{3}r^{\prime }\{\frac{3M\left(r^{\prime }\right)\cdot \left(r-r^{\prime }\right)}{{\left|r-r^{\prime }\right|}^{5}}\left(r-r^{\prime }\right)-\frac{M(r^{\prime })}{{\left|r-r^{\prime }\right|}^{3}}\}\;. \end{array}$$ (26)

For the linear magnetic materials, (Eq 26) is completed by the magnetostatics constitutive equation:

$$\begin{array}{c} M\left(r\right)=\frac{\chi (r)}{\mu_{0}\left(1+\chi \left(r\right)\right)}B\left(r\right)\;, \end{array}$$ (27)

which reduces to

$$\begin{array}{c} M\left(r\right)=\frac{\chi (r)}{\mu_{0}}B\left(r\right) \end{array}$$ (28)

for diamagnetic and paramagnetic substances whose susceptibility |*χ*| ≪ 1.

As in the MRI context ${\text{B}}_{0}≃B_{0}\hat{z}$, the $\hat{z}$ component of the field variation due to susceptibility inhomogeneities is therefore given by

$$\begin{array}{ccc} \Delta B_{z}\left(r\right) & = & \frac{B_{0}}{4\pi }\int_{V}d^{3}r^{\prime }\{\frac{3\chi \left(r^{\prime }\right)\cdot {\left(z-z^{\prime }\right)}^{2}}{{\left|r-r^{\prime }\right|}^{5}}-\frac{\chi (r^{\prime })}{{\left|r-r^{\prime }\right|}^{3}}\} \end{array}$$ (29)

$$\begin{array}{cc} = & B_{0}\cdot \left(\chi *G\right)\left(r\right)\;, \end{array}$$ (30)

where

$$\begin{array}{c} G\left(r\right)\equiv \frac{1}{4\pi }\cdot \frac{3z^{2}-r^{2}}{r^{5}}\;. \end{array}$$ (31)

As we acquire a spoiled GRE sequence, the phase associated to susceptibility (see (Eq 1)) is therefore given by

$$\begin{array}{c} \phi_{\chi }\left(r\right)=-\gamma_{n}T_{E}B_{0}\cdot \left(\chi *G\right)\left(r\right)\;. \end{array}$$ (32)

In order to extract *ϕ*_*χ*_(**r**) from the original wrapped phase *ψ*(**r**)

$$\begin{array}{c} \psi \left(r\right)=\phi \left(r\right)-2\pi \lfloor \frac{\phi (r)}{2\pi }\rfloor \end{array}$$ (33)

(which also depends on background magnetic field inhomogeneities, the imaginary coil sensitivity ℑ(*K*), RF-pulse design etc.), phase unwrapping was performed using the 3D-SRNCP algorithm [12], followed by the SHARP filter [13], which exploits the harmonicity of static magnetic fields within homogeneous media (∇^2^(*ϕ*−*ϕ*_*χ*_) = 0) [14]:

$$\begin{array}{c} \phi_{\chi }\left(r\right)=\text{SHARP}[\phi (r)]\;. \end{array}$$ (34)

The inversion of (Eq 32) requires a regularization of the inverse of *g*(**k**) ≡ FT[*G*(**r**)] (see [8, 15]), whence we finally obtain

$$\begin{array}{c} \chi \left(r\right)=-\frac{{\text{FT}}^{-1}[g_{\text{reg}}^{-1}\left(k\right)\cdot {\tilde{\phi }}_{\chi }\left(k\right)]}{\gamma_{n}T_{E}B_{0}}\;. \end{array}$$ (35)

### 2.7 *R*_2_-map

Once the *R*_1_- and the PD-maps are known, (Eq 6) makes it possible to derive the *R*_2_-map. From the following combination of Eqs (3), (5) and (6)

$$\begin{array}{c} \frac{\left|S_{0}\right|}{\left|K\right|M_{0}}=\frac{\left(1-E_{1}\right)\sin\theta \cdot E_{2}^{T_{E}/T_{R}}}{1-E_{1}\cos\theta -\left(E_{1}-\cos\theta \right)E_{2}^{2}}\;, \end{array}$$ (36)

if the acquired bSSFP scans are set with *T*_*E*_ = *T*_*R*_/2, it follows that

$$\begin{array}{c} a_{4}x^{4}+a_{1}x+a_{0}=0\;, \end{array}$$ (37)

where

$$\begin{array}{ccc} x & \equiv & \sqrt{E_{2}}\;, \end{array}$$ (38)

$$\begin{array}{ccc} a_{0} & \equiv & \frac{\left|S_{0}\right|}{\left|K\right|M_{0}}\left(E_{1}\cos\theta -1\right)\;, \end{array}$$ (39)

$$\begin{array}{ccc} a_{1} & \equiv & (1-E_{1})\sin\theta \;, \end{array}$$ (40)

$$\begin{array}{ccc} a_{4} & \equiv & \frac{\left|S_{0}\right|}{\left|K\right|M_{0}}\left(E_{1}-\cos\theta \right)\;. \end{array}$$ (41)

From the standard theory of the quartic equations it follows that the physically meaningful solution of (Eq 37) is given by

$$\begin{array}{c} x=\frac{1}{2}\sqrt{\frac{q}{S}-4S^{2}}-S\;, \end{array}$$ (42)

where

$$\begin{array}{ccc} q & \equiv & \frac{a_{1}}{a_{4}}\;, \end{array}$$ (43)

$$\begin{array}{ccc} S & \equiv & \frac{1}{2}\sqrt{\frac{1}{3a_{4}}(Q+\frac{\Delta_{0}}{Q})}\;, \end{array}$$ (44)

$$\begin{array}{ccc} Q & \equiv & \sqrt[{3}]{\frac{\Delta_{1}+\sqrt{\Delta_{1}^{2}-4\Delta_{0}^{3}}}{2}}\;, \end{array}$$ (45)

$$\begin{array}{ccc} \Delta_{0} & \equiv & 12a_{4}a_{0}\;, \end{array}$$ (46)

$$\begin{array}{ccc} \Delta_{1} & \equiv & 27a_{4}a_{1}^{2}\;. \end{array}$$ (47)

The transverse relaxation rate is then found from the expression

$$\begin{array}{c} R_{2}=-\frac{2\log x}{T_{R}}\;. \end{array}$$ (48)

## 3 Materials and Methods

### 3.1 Study design

**Fig 1:**
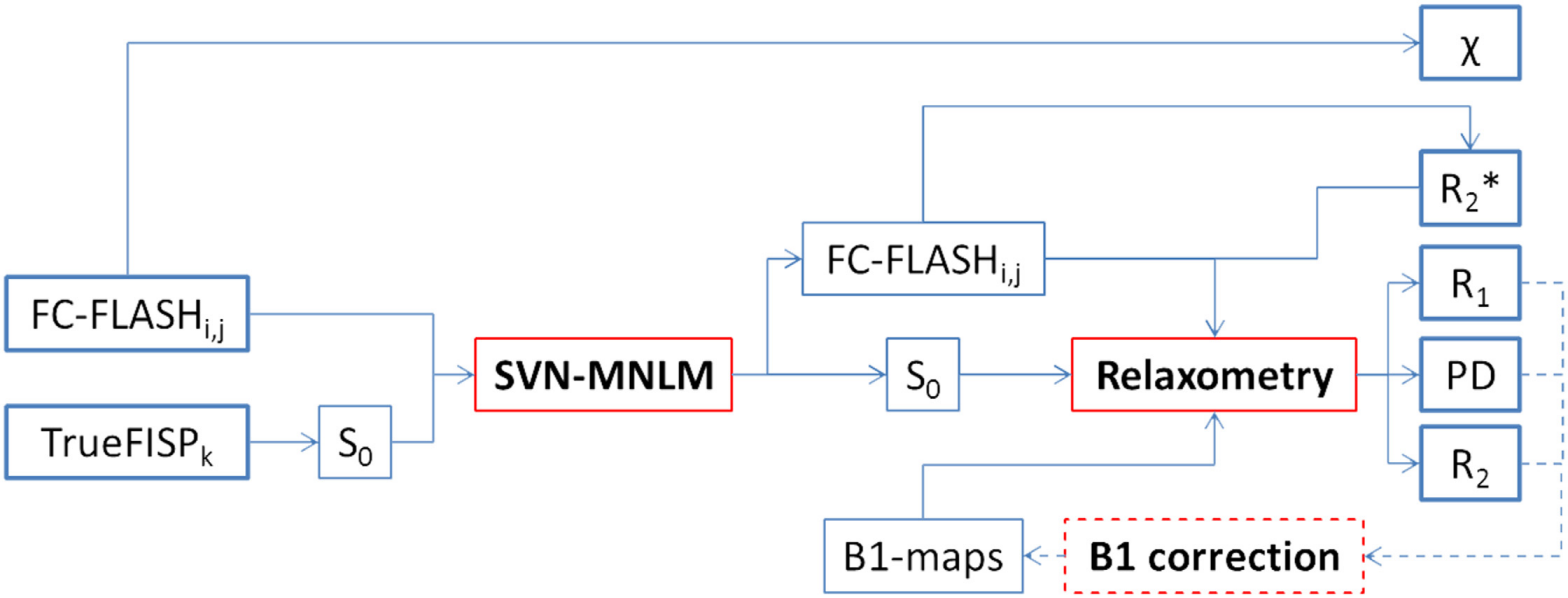
Schematic flowchart of the implemented algorithm. From FC-FLASH phase data the *χ*-map is directly derived (see §2.6). The TrueFISP collection is used to extract the band-free *S*_0_ dataset (see §2.2). Then, FC-FLASH and *S*_0_ series are denoised by the SVN-MNLM scheme (see §3.3). The $R_{2}^{*}$-map is computed from resulting FC-FLASH series (see §2.4). The introduction of an estimate of the *B*_1_-map (actually measured or simply guessed) allows for the extraction of the *R*_1_- (see §2.3), PD- (see §2.5) and *R*_2_- (see §2.7) maps. Depending on the reliability of the *B*_1_-map estimate, a *B*_1_-correction scheme may be iteratively applied (dashed lines—see §3.4).

We set up an experimental configuration to test the practical feasibility of the above general theoretical algorithm (schematically shown in Fig 1). It should be noted that, depending on the specific features of the available MR system, different strategies may be adopted.

First, we describe the acquisition protocol; then we present the image restoration algorithm we applied to increase the Signal to Noise Ratio (SNR) of the datasets. Further, as on our scanner there is no protocol for mapping *B*_1_ sensitivity profile, we describe the method developed to account for non-ideality of the $B_{1}^{\pm }$ profiles within the Field of View (FOV). Finally, we detail the way devised to estimate the overall reproducibility of the results.

### 3.2 Acquisition protocol

MR data were collected on a 3 T scanner (Trio, Siemens Medical Systems, Erlangen, Germany) with a volume transmitter coil and 8-channel head receiver coil. The “Carlo Romano” ethics committee for biomedical activities of “Federico II” University of Naples (Italy) specifically approved the study and the written informed consent form, which was signed by the subjects (one Healthy Control (HC) and one Relapsing-Remitting Multiple Sclerosis (MS) patient) undergoing the MR scan.

The 3D Steady-State sequences were acquired with a pseudo-axial orientation (along the anterior commissure-posterior commissure line) and shared the same FOV covering the whole brain (230 mm in anteroposterior direction; 194 mm in laterolateral direction; 166 mm in craniocaudal direction) with an in-plane resolution of 0.65 mm (laterolateral phase encoding direction) and a slice thickness of 1.3 mm (voxel size Δ*V* = 0.55 mm^3^).

We acquired 2 fully flow-compensated double-echo spoiled GRE (FC-FLASH) sequences with flip angles of 3° and 20°, repetition time of 28 ms, echo times of 7.63 ms and 22.14 ms, a GRAPPA factor of 2 and a bandwidth of 190 Hz/pixel (acquisition time: 2 × (4’ 46”)). For each echo, a magnitude/phase reconstruction was enabled, thus obtaining a total of 4 complex volume datasets.

Then, a set of 4 phase-cycled bSSFP (TrueFISP) sequences was acquired with resonance offset angle steps of *π*/2 (*i.e.* Δ*φ* ∈ {0, *π*/2, *π*, 3*π*/2}), flip angle of 10°, repetition time of 7.3 ms, echo time of 3.65 ms, a GRAPPA factor of 2 and a bandwidth of 252 Hz/pixel (acquisition time: 4 × (1’ 20”)). For each echo, a magnitude/phase reconstruction was enabled, thus obtaining a total of 4 complex volume datasets (*i.e.* one for each resonance offset angle).

It may be noted that, depending on the parameter subset of actual interest, this acquisition protocol may be shortened following the recipes shown in Table 1.

**Table 1:** Design guidelines for minimal acquisition protocols (second column) making it possible to reconstruct each parameter listed in the first column. The corresponding total acquisition time and the list of other maps that can be obtained for free are reported in the third and fourth columns, respectively.

Parameters	Required sequences	Acquisition time	Collateral maps
$R_{2}^{*}$, *χ*	FC-FLASH (Ernst FA)	4’ 46”	
*R*_1_, PD	FC-FLASH (FA = 3°)	9’ 32”	$R_{2}^{*}$, *χ*
	FC-FLASH (FA = 20°)		
*R*_2_	FC-FLASH (FA = 3°)	**14’ 52”**	$R_{2}^{*}$, *χ*, *R*_1_, PD
	FC-FLASH (FA = 20°)		
	TrueFISP		

### 3.3 Image restoration

In order to control the noise propagation in the relaxometry maps derived by inversion of the Bloch equations, the FC-FLASH and *S*_0_ image series were pre-processed with a novel *ad hoc* version of the Non-Local Means (NLM) filter, adapted for parallel MRI (see [16]) and multi-spectral images (see [17]) with an improved handling of the arbitrary noise power in each image component.

Spoiled GRE ({*S*_*i*,*j*_}) and bSSFP (*S*_0_) datasets were considered as a discrete version of a general multi-component image *X*: ℝ^*N*^ → ℝ^*M*^ (*N* = 3 and *M* = 5) with a bounded support Ω ⊂ ℝ^*N*^. In this sense, the Spatially-Varying-Noise Multi-component NLM (SVN-MNLM) is a class of endomorphisms of the image space, each identified by 2 parameters (*ρ* and ς), that act as follows:

$$\begin{array}{c} {\left[{\text{SVN}-\text{MNLM}}_{\rho ,\varsigma }(X)\right]}_{m}(\vec{x})=Y_{m}(\vec{x})=\frac{\int_{\Omega }W_{m}(\vec{x},\vec{y})X_{m}(\vec{y})d\vec{y}}{\int_{\Omega }W_{m}(\vec{x},\vec{y})d\vec{y}}\;, \end{array}$$ (49)

where

$$\begin{array}{c} W_{m}\left(\vec{x},\vec{y}\right)\equiv \exp[-\frac{d_{\rho }^{2}\left(\vec{x},\vec{y}\right)}{\varsigma^{2}}\cdot \frac{Q_{m}\left(\vec{x}\right)}{\sum_{l=1}^{M}Q_{l}\left(\vec{x}\right)}]\;, \end{array}$$ (50)

$$\begin{array}{c} d_{\rho }^{2}\left(\vec{x},\vec{y}\right)\equiv \sum_{m=1}^{M}\left\{\int_{{\mathbb{R}}^{N}}\frac{{\left|X_{m}\left(\vec{x}+\vec{t}\right)-X_{m}\left(\vec{y}+\vec{t}\right)\right|}^{2}}{\sigma_{m}^{2}\left(\vec{x}\right)+\sigma_{m}^{2}\left(\vec{y}\right)}\cdot \frac{\exp-\frac{\|\vec{t}{\|}^{2}}{2\rho^{2}}}{{\left(\sqrt{2\pi }\cdot \rho \right)}^{N}}d\vec{t}\right\}\;, \end{array}$$ (51)

$$Q_{m}(\vec{x})\equiv \frac{f_{\Omega }X_{m}^{2}(\vec{y})d\vec{y}}{\sigma_{m}^{2}(\vec{x})}\;,$$ (52)

$\rho ∼10\cdot \sqrt[{3}]{\Delta V}$ is the similarity radius, ς ∼ 1 is an adimensional constant to be manually tuned that determines the filter strength and $\sigma_{m}(\vec{x})$ is the standard deviation of noise of the *m* ^th^ image component at $\vec{x}\in \Omega$ (the noise power maps were estimated following [16]).

Due to the high computational complexity of the above scheme, a multi-Graphics Processing Unit (GPU) implementation of the NLM algorithm [18] was adapted to (Eq 49) and exploited for fast image processing.

### 3.4 $B_{1}^{\pm }$-inhomogeneity correction

The derivation of *R*_1_, PD and *R*_2_ maps in §2.3, §2.5 and §2.7 critically depends on the flip angle sensed by each voxel all-over the FOV. However, transmission coil design issues, the non-ideality of 3D slab profiles and the dielectric resonances, which are more prominent on high field scanners, may appreciably alter the $B_{1}^{+}$ homogeneity, thus resulting in a low frequency variation of the derived relaxometry parameters as we move from internal to peripheral brain regions. Moreover, the PD map also depends on the $B_{1}^{-}$ map of the receiver coil, while the other maps do not depend on the receiver coil sensitivity, as this constant factor is eliminated in the ratios of signal intensities in Eqs (20), (24) and (36).

Taking advantage of the large scale nature of the $B_{1}^{+}$ variation pattern, we adopted an information theory approach to remove the biases due to FA inhomogeneities. Essentially, a useful approximation of the peripheral FA decay is given by its second order Taylor logarithmic expansion about its stationary point ${\text{r}}_{0}^{+}$, modulated by a slab profile term:

$$\begin{array}{c} \text{FA}\left(r\right)={\text{FA}}_{0}\cdot \exp\{\frac{1}{2}{\left(r-r_{0}^{+}\right)}^{T}\cdot H^{+}\cdot \left(r-r_{0}^{+}\right)-k{\left|\zeta \right|}^{\xi }\} \end{array}$$ (53)

(*ζ* is the offset from the slab center in the slab select direction). As the Hessian tensor **H** ^+^ is symmetric, ${\text{r}}_{0}^{+}$ is a vector and *k*, *ξ* ∈ ℝ, a total of 11 free parameters need to be estimated in (Eq 53).

Similarly, the $B_{1}^{-}$ map can be estimated by a different second order Taylor logarithmic expansion (this time no slab profile affects the receiver coil sensitivity, thus reducing the number of free parameters to 9):

$$\begin{array}{c} K\left(r\right)=K_{0}\cdot \exp\{\frac{1}{2}{\left(r-r_{0}^{-}\right)}^{T}\cdot H^{-}\cdot \left(r-r_{0}^{-}\right)\}\;. \end{array}$$ (54)

Finally, the parameters are estimated by minimizing, with the Nelder-Mead method, the entropy of the 3D joint histogram related to *R*_1_, PD and *R*_2_ maps in low gradient regions of the maps.

The effectiveness of the obtained *B*_1_ inhomogeneity correction was evaluated by acquiring the full set of sequences on a cylindric homogeneous phantom (diameter: 18 cm; height: 30 cm) and computing the relative Full Width at Half Maximum (FWHM) of the *R*_1_ and *R*_2_ distributions reconstructed with and without the $B_{1}^{\pm }$-inhomogeneity correction.

### 3.5 Reproducibility estimation

To estimate the confidence interval of the proposed maps (taking into account image uncorrelated noise, variations in signal amplification of the MR scanner, tissue temperature fluctuations, etc.), each FC-FLASH was acquired twice and each TrueFISP three times in the HC during the same exam session, without moving the subject. Given the redundant set of complex datasets, an ensemble of 2^4^ (spoiled GRE) × 3 (*S*_0_) different complete relaxometry protocols was produced. Forty-eight different estimates of the relaxometry maps were thus derived and used to estimate the degree of reproducibility via mean and standard deviation maps.

### 3.6 Image processing and analysis

All data processing was performed using an in-house developed library for Matlab (MATLAB^®^ Release 2012b, The MathWorks, Inc., Natick, MA, USA), partly described in previous works [16–18], on a commercial workstation (Intel^®^ Core^™^ i7-3820 CPU @ 3.6 GHz; RAM 16 GB) equipped with 2 GPU boards (NVIDIA GeForce^®^ GTX 690).

The definition of the brain structures was qualitatively assessed in consensus by three experienced neuroradiologists, who were asked to indicate the specific diagnostic contribution provided by each map.

**Fig 2:**
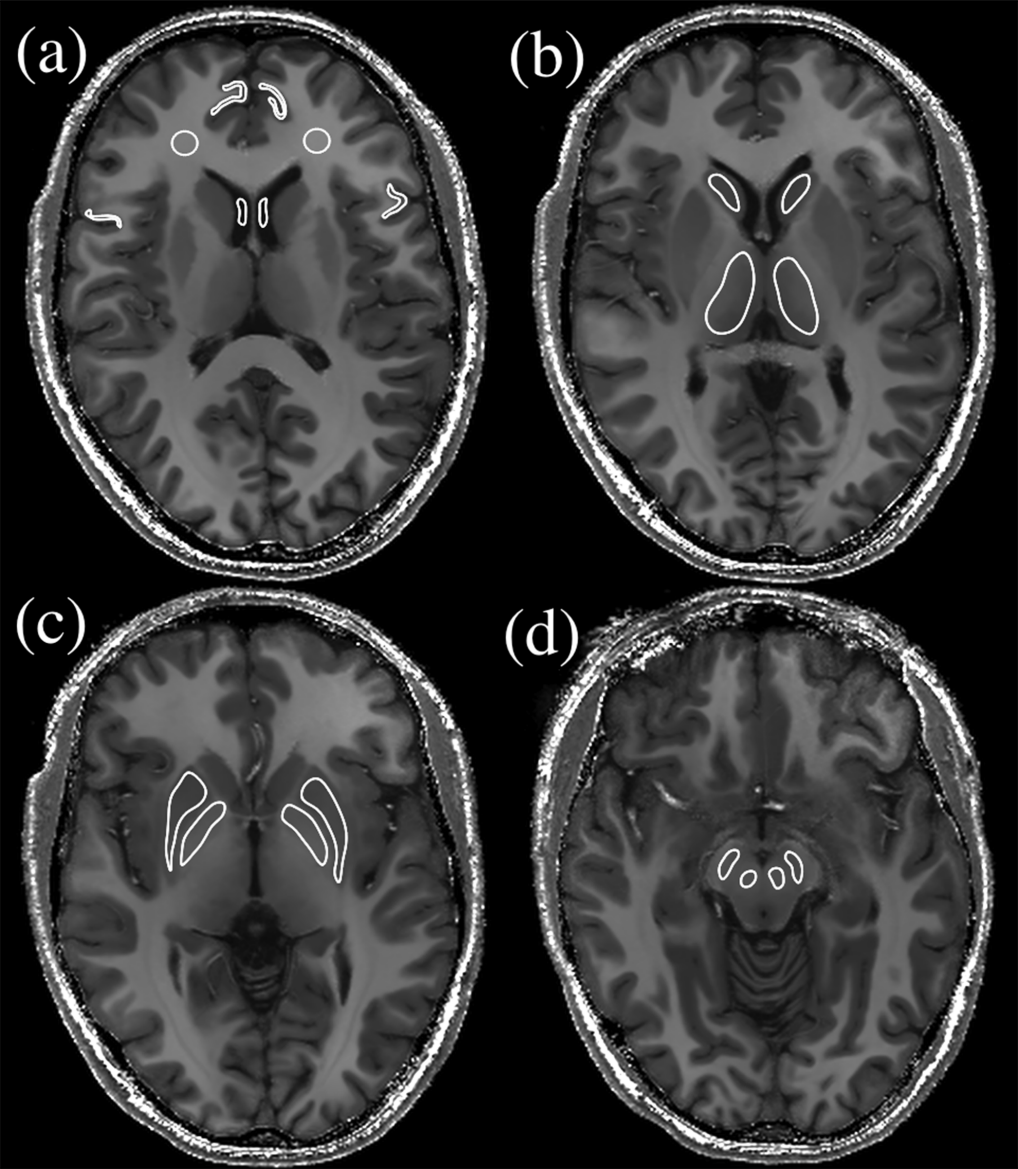
Axial brain slices at the 4 different levels showing the position of ROIs drawn for measurement of mean relaxometry values in selected brain structures: cortex, white matter and CSF (a); head of caudate and thalamus (b); globus pallidus and putamen (c); red nucleus and substantia nigra (d).

To further assess the capability of the quantitative parameters to distinguish between different intracranial structures, a set of bilateral ROIs (see Fig 2) was manually drawn on the *R*_1_-maps (and then automatically transferred to the other maps) of both the HC and MS subjects. ROIs were intentionally drawn well within the anatomical structures to avoid partial volume effects.

## 4 Results

### 4.1 Effectiveness of $B_{1}^{\pm }$-inhomogeneity correction

The test of the effectiveness of the proposed $B_{1}^{\pm }$-inhomogeneity correction on *R*_1_ and *R*_2_ is reported in Table 2.

**Table 2:** Percentual FWHM of the *R*_1_ and *R*_2_ distribution obtained without (pre) and with (post) $B_{1}^{\pm }$-inhomogeneity correction. For each case, we report the values for the entire phantom and for its eroded version (defined by a spherical structuring element with radius of 1 cm) simulating the brain without the skull.

		*R*_1_ FWHM		*R*_2_ FWHM	
		entire	eroded	entire	eroded
*B*_1_ correction	pre	36.47%	38.26%	21.28%	20.81%
	post	12.30%	8.95%	9.69%	8.71%

### 4.2 Map assessment

**Fig 3:**
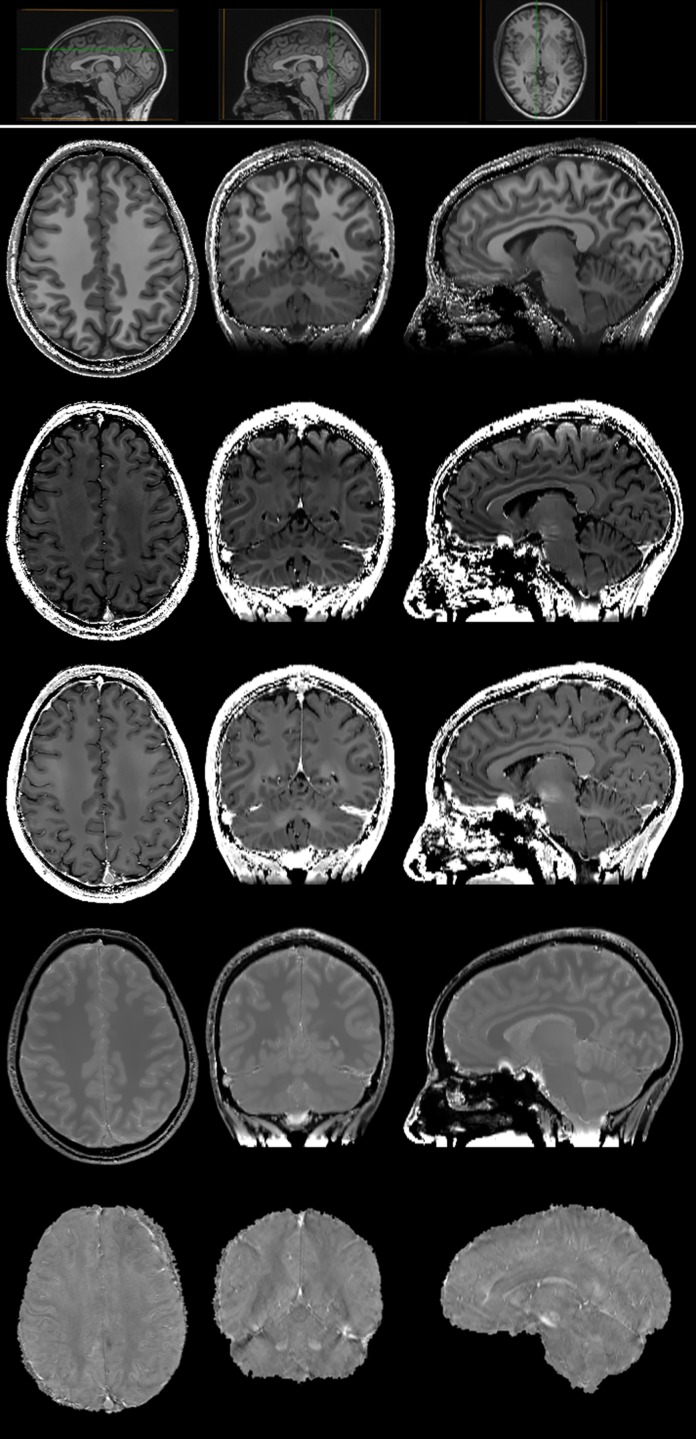
Axial (left), coronal (middle) and sagittal (right) brain slices of *R*_1_ ([0 ∼ 2] s^-1^), *R*_2_ ([0 ∼ 50] s^-1^), $R_{2}^{*}$ ([0 ∼ 50] s^-1^), PD ([0 ∼ 1] arbitrary units) and *χ* ([−300 ∼ 300] ppb) maps (from top to bottom) in a 43-year-old female HC. As coronal and sagittal images are derived by a multi-planar reconstruction of the original axial dataset, their in-plane resolution is 0.65 × 1.3 mm^2^, with a slice thickness of 0.65 mm. Small insets pointing out the position of the slices on a perpendicular plane are shown as anatomical reference in the upper row.

*R*_1_, *R*_2_, PD, $R_{2}^{*}$ and *χ* maps were obtained for each subject in about 30 minutes after completion of the acquisition. Examples of the maps for the 43-year-old female HC are displayed in Fig 3. Thanks to the acquired voxel size, the reconstructed datasets allow for reasonably good multi-planar reconstructions.

**Fig 4:**
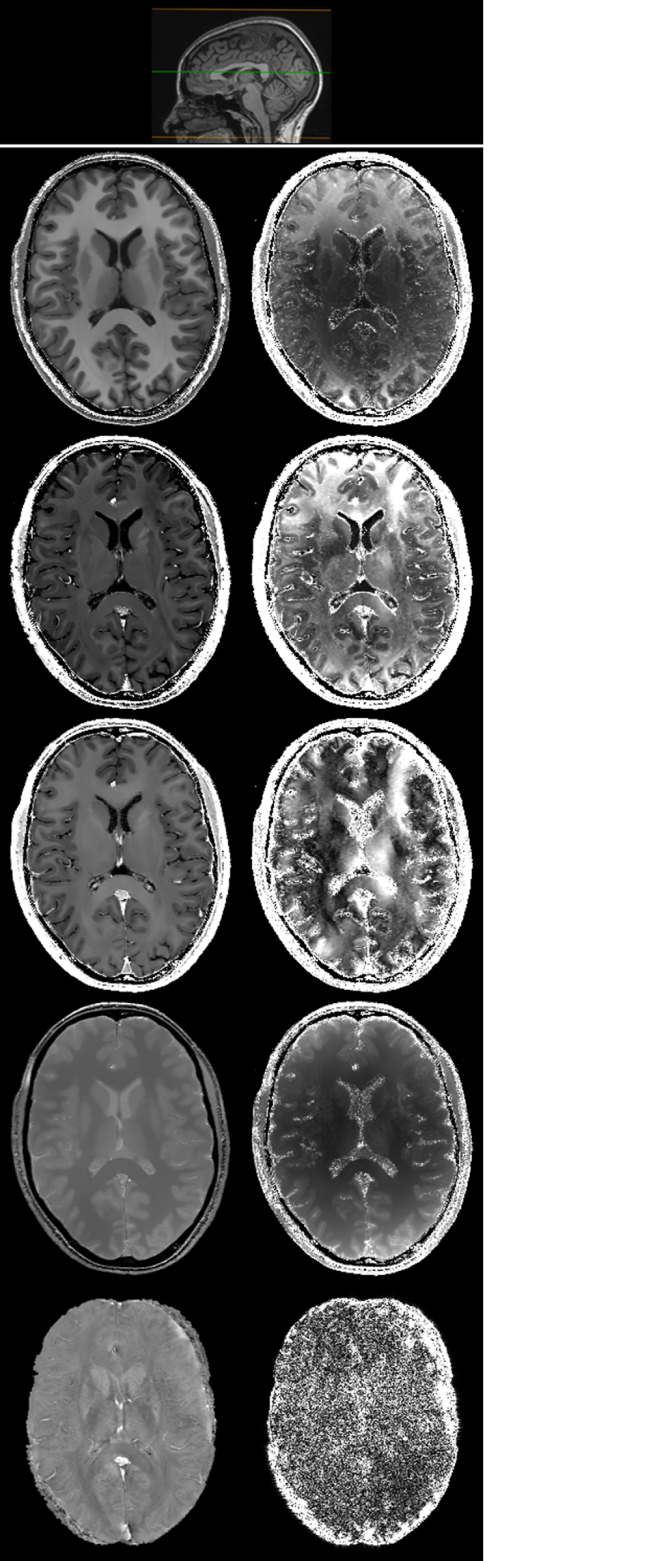
*R*_1_ ([0 ∼ 2] s^-1^), *R*_2_ ([0 ∼ 50] s^-1^), $R_{2}^{*}$ ([0 ∼ 50] s^-1^), PD ([0 ∼ 1] arbitrary units) and *χ* ([−300 ∼ 300] ppb) (from top to bottom) maps (left) and corresponding confidence interval (right) in a 43-year-old female HC, displayed with a markedly different (scale factor of 20) grayscale to highlight tiny differences in measure uncertainties. A small inset pointing out the position of the slices on a sagittal plane is shown as anatomical reference in the upper row.

In Fig 4 we report the mean quantitative maps and their corresponding voxel-by-voxel standard deviation maps, obtained as described in §3.5. On the whole, the standard deviation ranged from 2% to 4% of the quantitative absolute values, showing an excellent reproducibility of the map estimates.

### 4.3 Morphological evaluation

**Fig 5:**
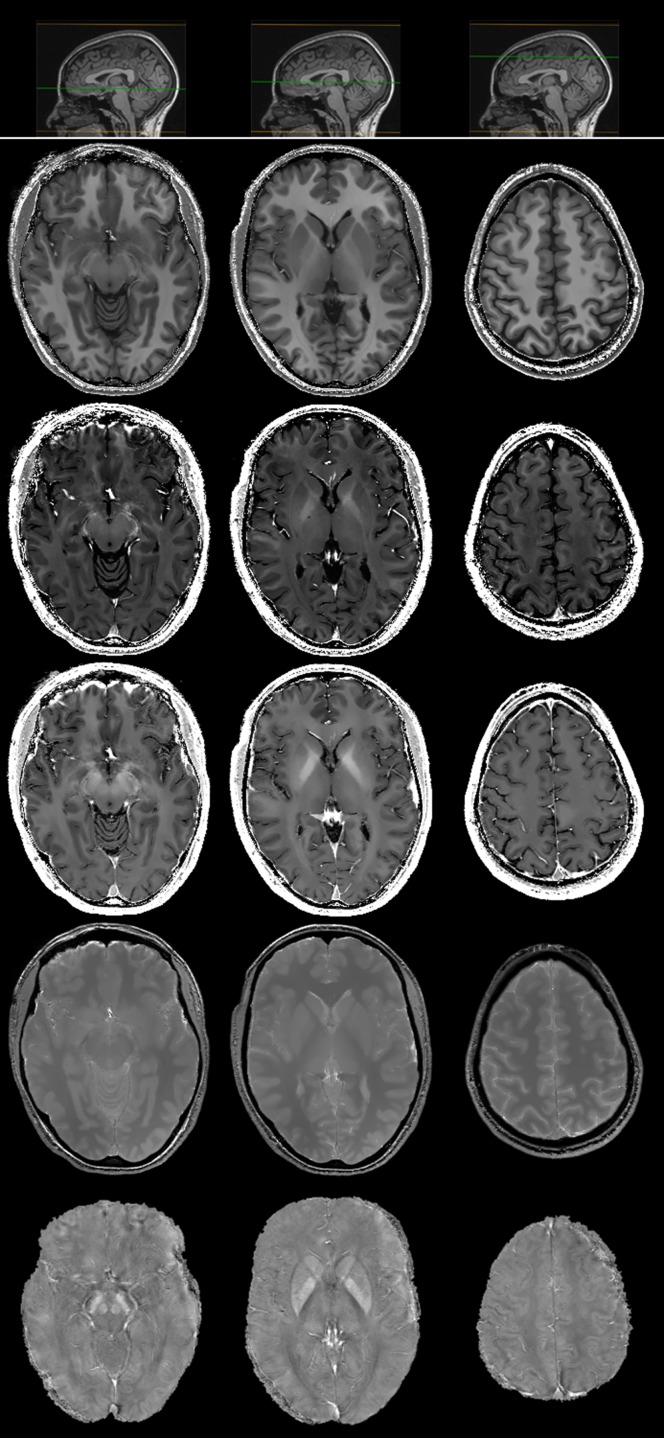
Axial brain slices at the level of midbrain (left), basal ganglia (middle) and fronto-parietal convexity (right) of *R*_1_ ([0 ∼ 2] s^-1^), *R*_2_ ([0 ∼ 50] s^-1^), $R_{2}^{*}$ ([0 ∼ 50] s^-1^), PD ([0 ∼ 1] arbitrary units) and *χ* ([−300 ∼ 300] ppb) maps (from top to bottom) in a 43-year-old female HC. Small insets pointing out the position of the slices on a sagittal plane are shown as anatomical reference in the upper row.

**Fig 6:**
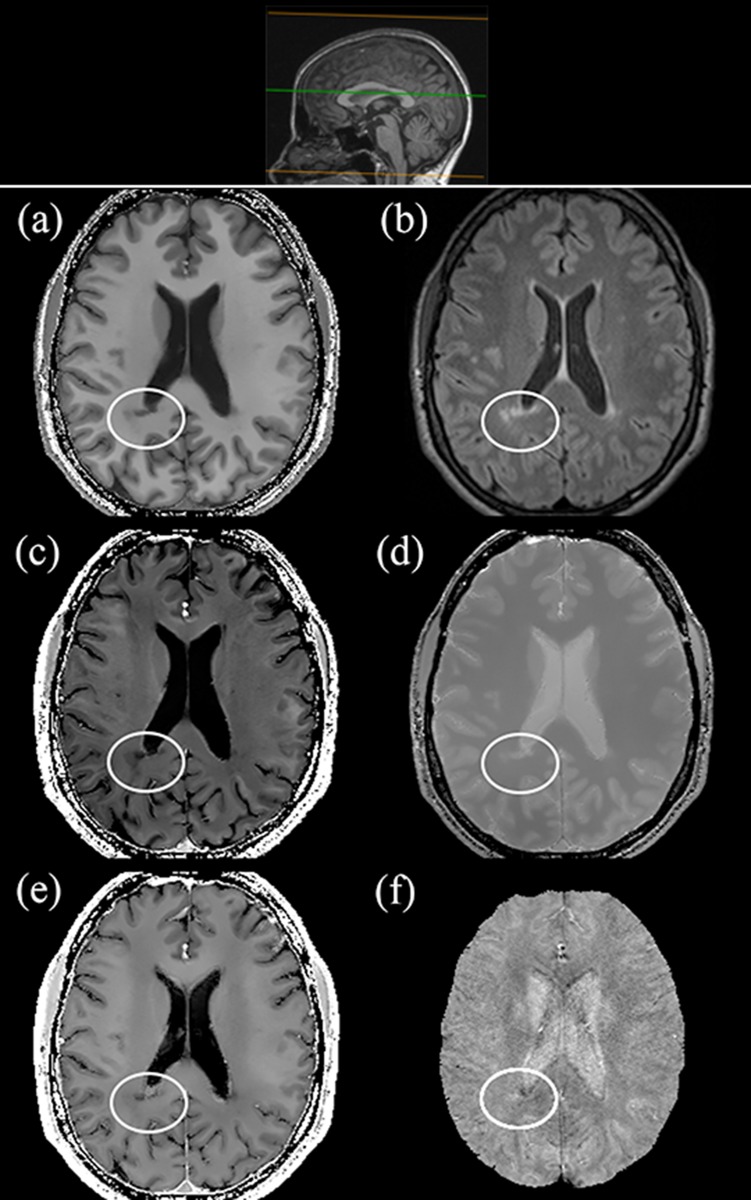
*R*_1_ (a), FLAIR (b), *R*_2_ (c), PD (d), $R_{2}^{*}$ (e) and *χ* (f) axial brain slices at the level of the lateral ventricles in a 45-year-old female MS patient (Expanded Disability Status Scale—EDSS: 3.5; disease duration: 7 years). A small inset pointing out the position of the slices on a sagittal plane is shown as anatomical reference in the upper row.

Fig 5 is an example of the maps assessed by the neuroradiologists in the qualitative analysis. In both HC and MS patient the *R*_1_-map was rated as the most representative of the brain anatomy, with excellent differentiation between grey (GM) and white (WM) matter. The $R_{2}^{*}$- and *χ*-maps clearly showed the subcortical structures known to induce significant local field inhomogeneities due to build-up of paramagnetic substances. In addition, in the MS patient, demyelinating lesions were clearly displayed on both *R*_1_- and *R*_2_-maps, even in critical areas, such as the periventricular WM (Fig 6).

The mean and standard deviation of the quantitative values of each structure and map are reported in Table 3.

**Table 3:** Relaxometry and susceptibility properties measured in selected brain locations. In each cell, ROI mean and standard deviation are reported.

	Structure	Area (mm^2^)	*R*_1_ (s^−1^)	*R*_2_ (s^−1^)	$R_{2}^{*}$ (s^−1^)	PD (A.U.)	*χ* ⋅ 10^9^
HC	RN	42	0.750 ± 0.009	17.24 ± 0.63	27.1 ± 1.0	0.811 ± 0.004	64 ± 21
	SN	63	0.727 ± 0.028	18.1 ± 1.1	29.3 ± 2.2	0.815 ± 0.013	155 ± 43
	Thalamus	486	0.689 ± 0.063	14.1 ± 1.0	20.1 ± 1.4	0.829 ± 0.029	8 ± 17
	Caudate	114	0.614 ± 0.023	13.91 ± 0.96	21.0 ± 1.5	0.853 ± 0.014	57 ± 14
	Putamen	325	0.645 ± 0.028	13.6 ± 1.6	22.8 ± 2.3	0.849 ± 0.013	50 ± 33
	Pallidus	230	0.797 ± 0.029	22.5 ± 1.5	35.6 ± 2.6	0.829 ± 0.020	153 ± 24
	WM	102	1.130 ± 0.010	12.11 ± 0.63	20.73 ± 0.54	0.676 ± 0.002	-10 ± 8
	GM (cortex)	91	0.648 ± 0.045	10.3 ± 1.0	14.9 ± 1.1	0.798 ± 0.016	16 ± 13
	CSF (ventricle)	35	0.179 ± 0.013	0.68 ± 0.45	2.9 ± 2.1	1.000 ± 0.028	26 ± 29
MS	RN	34	0.655 ± 0.018	21.0 ± 1.8	27.8 ± 1.0	0.831 ± 0.020	149 ± 26
	SN	47	0.725 ± 0.025	18.6 ± 1.1	29.0 ± 1.1	0.749 ± 0.015	132 ± 33
	Thalamus	463	0.644 ± 0.053	14.3 ± 1.1	20.8 ± 1.7	0.805 ± 0.024	6 ± 20
	Caudate	183	0.601 ± 0.030	13.2 ± 1.2	22.5 ± 2.2	0.811 ± 0.012	43 ± 22
	Putamen	215	0.653 ± 0.024	16.7 ± 1.2	26.2 ± 2.0	0.821 ± 0.014	74 ± 35
	Pallidus	194	0.779 ± 0.014	24.5 ± 1.9	38.9 ± 2.9	0.807 ± 0.011	157 ± 32
	WM	91	1.039 ± 0.012	12.25 ± 0.72	20.68 ± 0.87	0.651 ± 0.003	-18 ± 14
	GM (cortex)	93	0.625 ± 0.047	11.0 ± 1.9	16.6 ± 1.5	0.757 ± 0.017	11 ± 17
	CSF (ventricle)	40	0.158 ± 0.003	0.87 ± 0.58	2.7 ± 2.4	1.000 ± 0.013	24 ± 25

PD values are expressed in arbitrary units (A.U.). RN = Red Nucleus; SN = Substantia Nigra; Caudate = Head of Caudate Nucleus; Pallidus = Globus Pallidus; WM = White Matter; GM = Grey Matter; CSF = Cerebrospinal Fluid.

## 5 Discussion

We have described a 3D acquisition protocol that yields multiple quantitative parametric maps (*R*_1_, *R*_2_, $R_{2}^{*}$, PD and *χ*) based on the relaxometric properties of brain tissues.

Image quantitation in MRI has been widely studied in the last decades, with particular regard to tissue relaxometry, and almost any subset of the quantitative parameters obtained with our approach has been the subject of at least one study [1, 8, 9, 19]. However, a great majority of the proposed schemes largely rely on 2D acquisition sequences [3, 20–24], which, in this context, may be detrimental for at least two reasons. First, SAR issues limit the slice thickness to values considerably larger than the voxel thickness achievable in 3D acquisitions. Second, and most important, voxel intensities reconstructed from 2D acquisitions derive from the integration of isochromat signals over a non-ideal slice profile. Their Bloch equations in fact evolve according to flip angles that greatly vary over each voxel, unlike 3D sequences, in which intra-voxel flip angle variation is usually small compared to its mean value [9]. Therefore, 2D sequences usually lead to biases in *R*_1_ and *R*_2_ calculations, which are particularly hard to estimate if the RF-pulse is not completely known or for sequences leading to entangled relaxometry equations (e.g., any type of SSFP [25]).

Conversely, other groups have adopted 3D acquisition strategies to recover *R*_1_- and $R_{2}^{*}$-maps of the brain [6, 23], but not the quantitation of pure transverse relaxation rate (*R*_2_) or susceptibility (*χ*). These may provide useful information when *R*_1_ and $R_{2}^{*}$ fail to provide adequate information or contrast to reveal the pathophysiological conditions of specific areas (e.g. in presence of high susceptibility gradients near petrous bones, nasal sinuses, etc. that cause an $R_{2}^{*}$ enhancement uncorrelated to any tissue alteration).

Recently, some 3D approaches have been proposed to derive *R*_1_- and *R*_2_-maps based on different modes of unbalanced SSFP signals [5, 7], with promising results in the study of joints, cartilage and, in general, of the musculoskeletal system [26, 27]. However, 3D unbalanced SSFP sequences are known to be highly sensitive to pulsatile flow of fluid with relatively long *T*_2_, thus making the brain study unfeasible, due to CSF pulsation, unless switching to 2D [28].

In this respect, the family of DESPO methods [2, 29–31], while neglecting $R_{2}^{*}$- and *χ*-maps, do provide a full 3D high SNR *R*_1_- and *R*_2_-maps [2], using sequences poorly prone to motion artifacts (spoiled GRE and bSSFP [32, 33]). Moreover, the numerical approach described in [31] is able to subtract the banding artifacts due to *B*_0_ inhomogeneities sensitivity of bSSFP. However, some drawbacks of DESPOT2 may raise crucial issues even if $R_{2}^{*}$ and susceptibility information are not of interest. In particular, even if bSSFP sequences are usually quite insensitive to motion, when flip angle gets close to 90 degrees, CSF pulsatile flow may cause some periventricular hyperintensities due to ghosting artifacts in phase encoding directions. This, in turn, leads to a biased estimate of *R*_2_ in the periventricular WM that may mimic, for example, demyelinating lesions. Moreover, the same high flip angle values prescribed for at least one bSSFP of the DESPOT2 protocol are likely to exceed SAR constraints, especially if a short *T*_*R*_ is desired to shorten total acquisition time.

The scheme we propose is meant to solve the above mentioned issues. As DESPO, our approach entirely relies on widely available 3D acquisition sequences. Moreover, it allows for the quantitation of 5 independent parameters and gets rid of the sensitivity to *B*_0_ inhomogeneity by means of a fully analytical solution (thus speeding up the computation step). As for the $B_{1}^{\pm }$ inhomogeneity dependence, one can completely remove it using a measured *B*_1_ field map, if an *ad hoc* protocol is available on the scanner; otherwise, as shown in §3.4 and §4.1, an information theory approach can be exploited to largely recover the map homogeneity. Furthermore, a judicious use of the Bloch equations for the acquired MR signals enabled us to skip the acquisition of high flip angle bSSFPs, thus limiting the required acquisition time and avoiding both SAR issues and CSF pulsation artifacts.

As for the quantitation of brain tissue parameters, the results are encouraging. From a pure theoretical point of view, it is noteworthy that the well known physical constraints on relaxation rates ($R_{1}<R_{2}<R_{2}^{*}$) are satisfied for any structure/tissue analyzed in Table 3, despite the highly uncorrelated acquisition strategies we adopted to estimate the associated maps. A comparison of ours with the corresponding estimated values present in the literature also confirms the reliability of the ranges we found in the different compartments. In particular, a good agreement is found for longitudinal relaxation (GM: 0.609 ± 0.008 s^-1^ in [6] or 0.622 ± 0.043 s^-1^ in [34]; WM: 1.036 ± 0.036 s^-1^ in [6] or 1.190 ± 0.071 s^-1^ in [34]; caudate nucleus: 0.683 ± 0.022 s^-1^ in [6] or 0.719 ± 0.025 s^-1^ in [34]) and transverse FID (GM: 15.2 ± 0.4 s^-1^; WM: 21.0 ± 0.8 s^-1^; caudate nucleus: 18.2 ± 1.2 s^-1^ in [6]) rates measured at 3 T. As for *R*_2_ values, which are usually less sensitive to *B*_0_ magnitude than *R*_1_ and $R_{2}^{*}$, 3D measures performed at 1.5 T substantially match our findings (GM: 10.20 ± 0.73 s^-1^; WM: 18.5 ± 1.4 s^-1^; caudate nucleus: 11.24 ± 0.76 s^-1^ in [29]), except for an unusually high value found in WM by [29], which significatively differs also from several other 2D measures [3, 9, 20, 35]. Also, our PD estimates are in line with those reported in the literature (GM/CSF: 0.796 ± 0.078 in [20] or 0.844 ± 0.019 in [6]; WM/CSF: 0.742 ± 0.070 in [20] or 0.683 ± 0.006 in [6]; caudate nucleus/CSF: 0.851 ± 0.084 in [20] or 0.827 ± 0.016 in [6]). Finally, our *χ* measures fit well with the results of previous studies (GM: 16 ± 10 parts-per-billion (ppb); WM: −18 ± 9 ppb; globus pallidus: 200 ± 40 ppb; putamen: 70 ± 20 ppb; substantia nigra: 200 ± 60 ppb; red nucleus 90 ± 30 ppb in [13]). However, it should be borne in mind that plenty of variable factors (such as inter-subject variability, *B*_0_, voxel size, ROI definitions, scanner-specific biases, 2D or 3D imaging modality, etc.) may alter the qMRI results and, as such, should recommend caution in interpreting differences or similarities [6].

Visual inspection of our parametric maps also showed the expected features of normal structures (Fig 5) and of pathological tissue changes, such as demyelinating lesions (Fig 6).

We are aware that the present study is not without limitations. Due to the absence of a protocol for mapping the RF-transmission profile and the receiver coil sensitivity, we were forced to enable the $B_{1}^{\pm }$-inhomogeneity correction introduced in §3.4, which can greatly limit the map inhomogeneities, but not completely compensate for it. Moreover, the underlying assumption of the present version of our scheme is that both longitudinal and transverse relaxations follow a mono-exponential recovery of the equilibrium condition. Even if this is a common assumption in relaxometry studies, there is evidence that WM shows multiple relaxation components, each with its own relaxing water pool [30]. In this respect, further theoretical and clinical analysis will be devoted to extend the present scheme to a multicomponent generalization.

In conclusion, we found a fully analytical solution to a 5 parameter qMRI problem that enables the reconstruction of 3D brain datasets with submillimiter resolution in a clinically feasible acquisition time (less than 15 minutes in our experimental setup). Our maps do not suffer from many of the issues affecting previously reported strategies, and allow for a more accurate characterization of brain structures. We believe this will trigger a convenient access to unconventional ways of studying a large spectrum of pathophysiological conditions.
